# Molecular Tools for *Lynx* spp. qPCR Identification and STR-Based Individual Identification of Eurasian Lynx (*Lynx lynx*) in Forensic Casework

**DOI:** 10.3390/mps8030047

**Published:** 2025-05-02

**Authors:** Karolina Mahlerová, Johana Alaverdyan, Lenka Vaňková, Daniel Vaněk

**Affiliations:** 1 Institute for Environmental Studies, Charles University, 128 01 Prague, Czech Republic; johana.alaverdyan@suro.cz (J.A.); lenka.vankova@fdnas.cz (L.V.); 2 Forensic DNA Service, 170 00 Prague, Czech Republic; 3 Department of Ecology, Faculty of Environmental Sciences, Czech University of Life Sciences, 165 00 Prague, Czech Republic; 4 Department of Legal Forensic Medicine, Bulovka University Hospital, 180 00 Prague, Czech Republic; 5 Department of Forensic Medicine, Second Faculty of Medicine, Charles University, 120 00 Prague, Czech Republic

**Keywords:** wildlife crime, wildlife trade, Feliformia, Felidae, *Cyt b*

## Abstract

The Eurasian lynx (*Lynx lynx*) is listed in CITES Appendix II and is protected under the Bern Convention and the EU Habitats Directive, yet it remains a frequent target of wildlife crime, highlighting the urgent need for reliable identification methods. This study focuses on determination and DNA quantification of the *Lynx* spp. using quantitative real-time PCR (qPCR). The *Llynx Qplex* quantification multiplex system effectively distinguishes *Lynx* spp. from other Feliformia species by targeting mitochondrial and nuclear markers. Additionally, we present the results of the developmental validation of the *Llyn STRplex* system for individual identification and databasing using six STR loci. This study followed ISFG recommendations for non-human DNA testing and developmental validation guidelines. Both systems demonstrate high sensitivity (5 pg genomic DNA for *Llynx Qplex* and 30 pg of mtDNA for *Llyn STRplex*) and high specificity to *Lynx* spp., confirmed by testing against 16 related Feliformia species. Robustness was evaluated, showing sensitivity to temperature variation, and both repeatability and reproducibility were successfully tested across replicates and conditions. Given that forensic casework often involves degraded and limited biological material, molecular tools must be both sensitive and specific to ensure accurate results. Developing precise and efficient tools is essential for supporting investigations of wildlife crime involving the Eurasian lynx, as well as efforts aimed at conserving the species.

## 1. Introduction

Wildlife crime has become a severe global issue, particularly affecting endangered species. The need for DNA-based animal species and individual identification in forensic casework increased significantly when wildlife crime gained prominence for law enforcement agencies. The main driving force for the expansion of non-human DNA testing is the growing connection between organized crime and wildlife crime [1,2,3]. The first scientific studies on animal and plant DNA identification appeared in the early years of identification genetics, even if the studies addressed non-forensic areas like species identification of tuna fish [4], phylogeny [5,6,7], conservation efforts [8], relationship [9,10], evolutionary studies [11,12,13], and paleobiology [14]. One of the first studies that dealt with the museum specimen of extinct zebra quagga [15] paved the road of DNA typing for species identification. The studies from the early 1990s identified the possibilities for the field of forensic studies [16,17,18]. Cases of disputed paternity of dogs solved by DNA fingerprinting were described in 1991 [19], the plant DNA analysis used as a piece of evidence was published in 1993 [20], and forensic tracing of horse identities was reported in 1996 [21]. However, the increased use of non-human DNA typing for forensic purposes started hand-in-hand with the availability of typing kits [22,23] and developmental and validation studies aiming at animal barcoding using mitochondrial DNA markers [23,24]. Even though human and non-human DNA typing for forensic purposes has many common features, it has been necessary to set criteria for this specific field. Budowle et al. created the first set of comprehensive guidelines [25]; their work was followed by the recommendations produced by ISFG in 2011 [26]. Some current studies on individual identification of animals are driven by the demands of local or international enforcement agencies. Illegal trade of rhinoceros horns can be traced using assays and a database originated in South Africa [27,28], illegal trade of elephant ivory can be successfully monitored using DNA-based tools developed by the group of S. K. Wasser [29,30,31,32], and illegal trade of Traditional Chinese medicine derived from Pantherinae body parts can be uncovered by procedures developed in the Czech Republic [33,34,35,36]. Another example of research on public demand are typing systems for European roe deer [37], bears [38,39], or wolves and dogs [40,41]. Accurate identification is crucial for species conservation and law enforcement efforts to combat wildlife crime.

DNA-based forensic techniques offer a robust solution, enabling species determination and individual identification. These methods commonly rely on barcoding (e.g., *Cytochrome c oxidase subunit I*, *Cytochrome b*) and short tandem repeat (STR) polymorphisms [35]. STRs have the dominant position in genetic monitoring of populations [42]. In the case of *Lynx* spp., STR-based approaches have been widely applied. Genetic monitoring of *Lynx lynx* using STR loci has been applied to estimate population size, inbreeding, sex ratio, and kinship in Scandinavia [43,44,45] and Central Europe [46,47]; *Lynx canadensis* [45] and *Lynx rufus* in Northern America [48]; and *Lynx pardinus* in Spain [49]. To ensure the repeatability and accuracy of STR analyses, precise measurement of DNA concentration is essential. Accurate quantification ensures that optimal DNA amounts are used in amplification reactions, minimizing variability and maximizing the reliability of results. The proper quantification of DNA extracted from forensic evidence is a necessary step following the extraction, as the subsequent STR genotyping requires an optimal input quantity. This step is particularly critical when working with suboptimal samples, including low-quality or low-quantity samples, such as those collected non-invasively from hair or fecal samples [50,51]. The most commonly used techniques for DNA quantification include UV spectrophotometry (e.g., NanoDrop instruments, ThermoFisher Scientific, Waltham, MA, USA), fluorometry (e.g., Qubit, Life Technologies, Carlsbad, CA, USA), gel electrophoresis, and qPCR using SYBR Green. However, these methods often suffer from insufficient sensitivity, and the precision of DNA concentration measurements may be compromised due to under- or overestimation. Additionally, they are generally unable to distinguish between intact and degraded DNA and often exhibit low specificity [52,53].

The Eurasian lynx (*Lynx lynx*) is a species strongly affected by wildlife crime, and its population is declining due to habitat fragmentation, habitat loss, and poaching [54,55]. Historically distributed across the Eurasian region [56], this species is now listed under CITES Appendix II and is protected by the Bern Convention and the EU Habitats Directive (Habitat Directive 92/43/EEC; Bern Convention). Despite these protections and gradually favorable public attitudes, the Eurasian lynx remains a frequent target of wildlife crime, highlighting the urgent need for reliable identification methods [57].

In this paper, we present a newly designed molecular system for *Lynx* spp. determination and DNA quantification based on real-time polymerase chain reaction (qPCR), including artificial internal positive control. The qPCR system is combined with a multiplex STR system that includes di-, tri-, and tetranucleotide repeats and integrates sex determination using the Amelogenin gene [45,58,59]. This system allows for *Lynx* spp. identification and individual identification of *Lynx* spp. from tested samples, offering a valuable tool for forensic science and wildlife conservation.

## 2. Materials Studied, Methods, Techniques

### 2.1. Specimens Used for the Analyses

The quantification and individual identification system was tested on 16 unrelated *Lynx* spp. individuals. Fecal samples were obtained from Zoological Gardens in the Czech Republic. The tissue samples were obtained from dead animals (traffic accident, tanned hide). The sampling process, therefore, did not cause any harm or trauma to living animals. The protection of animals used for scientific purposes, as stated by the Directive 2010/63/EU of the European Parliament and of the Council of 22 September 2010, was fully respected. Fecal samples were collected and stored in DNA/RNA Shield Fecal Collection Tubes (Zymo Research, Irvine, CA, USA) prior to DNA extraction. DNA was extracted from fecal samples using the Quick-DNA Fecal/Soil Microbe Miniprep Kit (Zymo Research, Irvine, CA, USA), and tissue samples were extracted using the Quick-DNA Microprep Plus Kit (Zymo Research, Irvine, CA, USA) following the manufacturer’s protocols.

### 2.2. Quantification System and Species Identification

The extracted DNA was quantified using the *Llynx Qplex* quantification system, using qPCR (QuantStudio™ 5 Real-Time PCR System, ThermoFisher Scientific) and utilizing TaqMan probes and four standards (S1: 46 ng/μL mtDNA, 0.06 ng/μL nDNA; S2: 9.2 ng/μL mtDNA, 0.012 ng/μL nDNA, S3: 1.84 ng/μL mtDNA, 0.0024 ng/μL nDNA; S4: 0.368 ng/μL mtDNA, 0.00048 ng/μL nDNA). The system targeted the following sequences: an mtDNA fragment of Cytochrome b (*Cyt b*) (139 bp) (mtDNA) specific to *Lynx* spp., a fragment of nuclear proteolipid protein (PLP) (132 bp) (nDNA) specific to Feliformia, and the artificial Internal Positive Control (IPC) (261 bp) (Table 1). The primers were designed using Primer-BLAST [60], and probes were designed using Primer Express v3.0.1 (ThermoFisher Scientific, USA). All primers were analyzed together using an online software, Multiple Primer Analyzer (ThermoFisher Scientific, USA), for primer dimer detection prior to further testing. The quantification reaction consisted of a total volume of 10 μL (5 μL of 2× TaqMan Multiplex Master Mix (ThermoFisher Scientific, USA), 0.5 μL of 20× qLynx mtDNA Assay mix, 0.5 μL of 20× qLynx nDNA Assay mix, 0.5 μL of 20× qLynx IPC, 1 μL of IPC 0.1 pg/μL, 2.5 μL of DNase/RNase-Free Water (Zymo Research), and 1 μL of DNA template) under the following cycling conditions: 95 °C 20 s; 50× 95 °C 10 s; 60 °C 25 s. The results were analyzed using QuantStudioTM Design & Analysis Software v1.5.2 (ThermoFisher Scientific, USA). Each run included positive control (a selected individual of *Lynx lynx* used consistently across all test runs) and a negative control consisting of DNase/RNase-Free Water (Zymo Research).

### 2.3. STR System for Individual Identification

Individual identification was conducted using *Llyn STRplex*, which contains five di-, tri-, or tetranucleotide STR loci; additionally, sex determination was integrated using the Amelogenin gene (Table 2). STRs were amplified using fluorescently labeled primers with standard PCR in a total volume of 12.5 μL (1.25 μL of Gold Star 10x buffer (Promega, Madison, WI, USA), 0.25 μL of AmpliTaq Gold DNA Polymerase (ThermoFisher Scientific, USA), 1.25 μL of 10x *Llyn STRplex* Primer Mix (Table 2), template DNA (~30–50 pg of nDNA), and DNase/RNase-Free Water (Zymo Research) to a total volume of 12.5 μL) under the following cycling conditions: initial denaturation at 95 °C for 11 min, followed by 32 cycles of 94 °C for 30 s, 55 °C for 1 min 10 s, and 72 °C for 90 s, with a final extension at 72 °C for 60 min followed by 60 °C for 60 min. Fragment analysis was conducted via capillary electrophoresis with the SeqStudio™ 3200 Genetic Analyzer System (ThermoFisher Scientific, USA) using the following mix of 12 μL of Hi-Di Formamide and 0.3 μL of LIZ600size standard (ThermoFisher Scientific, USA) mixed with 1 μL of the PCR product Under following settings: Size standard GS600LIZ, Dye Set G5 (DS-33), Frag analyses 1200 sec. The data were subsequently analyzed using the genotyping software GeneMapper v5 (ThermoFisher Scientific, USA). Each run included positive control (a selected individual of *Lynx lynx* used consistently across all test runs) and a negative control consisting of DNase/RNase-Free Water (Zymo Research).

## 3. Results

**Figure 1:**
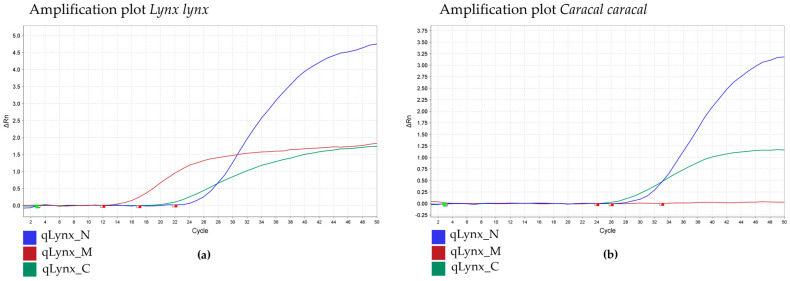
The qPCR quantitation system *Llynx Qplex* targeting genus-specific mtDNA (red), nuclear DNA (blue), and Internal Positive Control (green): (**a**) Extracted DNA from *Lynx lynx*. (**b**) Extracted DNA from *Caracal caracal*.

**Figure 2:**
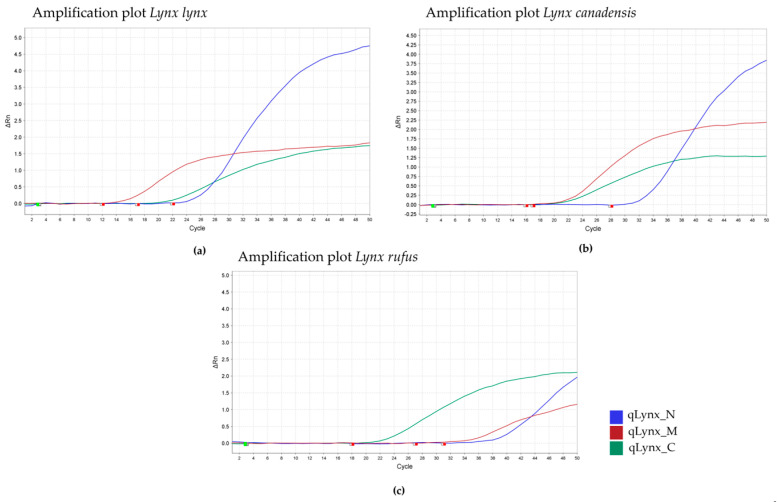
Specificity of *Llyn Qplex* to *Lynx* spp.—*Lynx lynx* (**a**), *L. canadensis* (**b**), and *L. rufus* (**c**).

**Figure 3:**
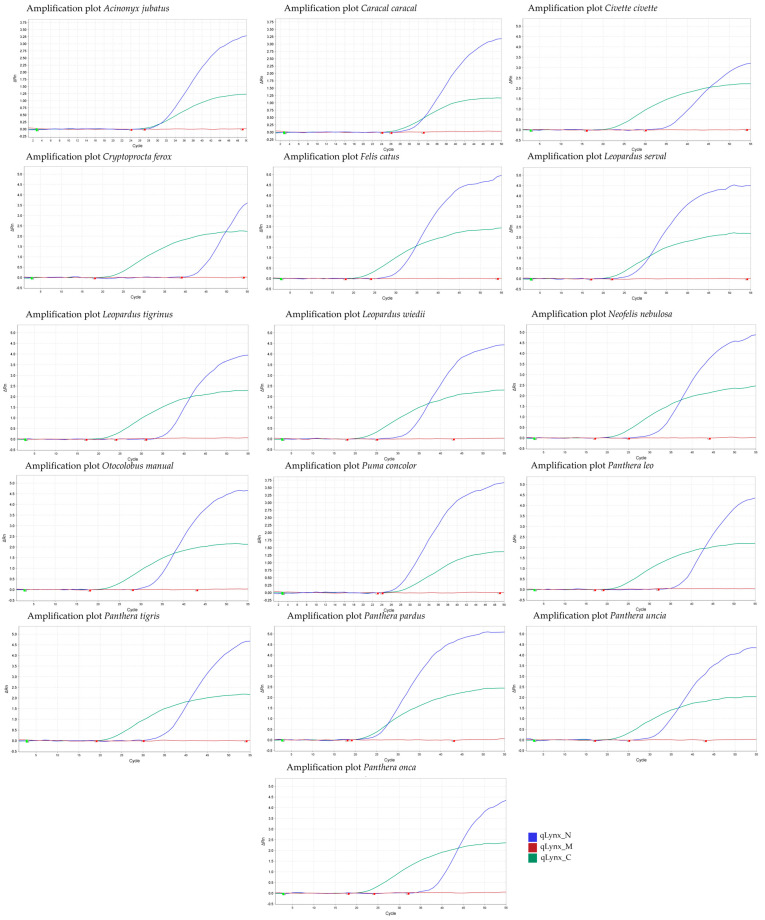
Amplification plot of 16 species of Feliformia demonstrates the specificity of the *Llynx Qplex Acinonyx jubatus*, *Caracal caracal*, *Civette civette*, *Cryptoprocta ferox*, *Felis catus*, *Leopardus serval*, *L. tigrinus*, *L. wiedii*, *Neofelis nebulosa*, *Otocolobus manual*, *Puma concolor*, *Panthera leo*, *P. tigris*, *P. pardus*, *P. uncia*, and *P. onca* (~10 ng/µL).

The identification of *Lynx* spp. in a sample was performed using qPCR. The presence of an amplification curve for the *Cyt b* fragment indicates the presence of *Lynx* spp. in the sample (Figure 1 and Figure 2). The *Llynx Qplax* has been tested on 16 closely related taxa to test the specificity of the mtDNA marker (Figure 3).

**Figure 4:**
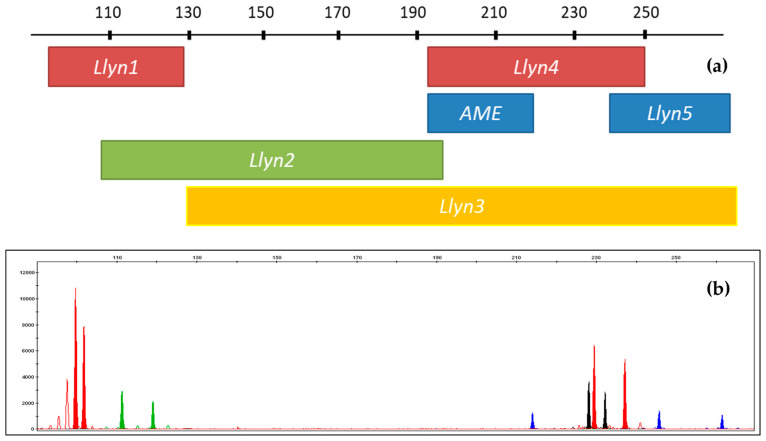
STR-multiplexed *Llyn STRplex* for DNA-based individual identification of *Lynx lynx*: (**a**) schematic representation of the analyzed loci; (**b**) results obtained from the capillary electrophoresis.

**Figure 5:**
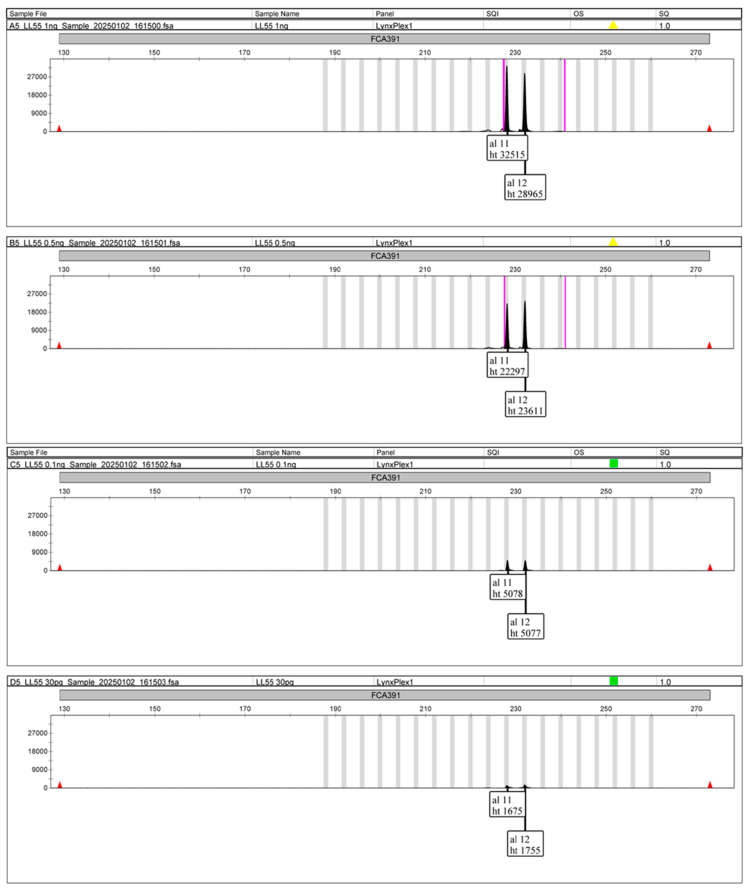
The results of the sensitivity study (from top to bottom—1 ng, 0.5 ng, 0.1 ng, and 30 pg of nDNA)—shown on FCA391 (*alias Llyn 3*).

The optimal concentration for STR-based individual analysis was determined to be ~30–50 pg of nDNA. Individual identification and sex determination of lynxes were conducted based on 5-variable STR loci and a sex determination system (Figure 4). The results of the STR typing are shown in Table 3 and Figure 5.

## 4. Validation

### 4.1. Llynx Qplex

Specificity, sensitivity, robustness, repeatability, and reproducibility were assessed following the protocol for the *Llynx Qplex* assay described in the Materials and Methods Section 2.2.

Specificity: The Llynx Qplex quantification assay was tested on 16 phylogenetically related species belonging to Feliformia (Acinonyx jubatus, Caracal caracal, Civette civette, Cryptoprocta ferox, Felis catus, Leopardus serval, L. tigrinus, L. wiedii, Neofelis nebulosa, Otocolobus manual, Puma concolor, Panthera leo, P. tigris, P. pardus, P. uncia, P. onca) and four outgroup species—Bos taurus, Canis lupus familiaris, Homo sapiens, Ursus arctos. DNA extracts from all tested species were diluted to 10 ng/µL and analyzed using the Llynx Qplex assay. A positive control (Lynx lynx DNA; 10 ng/µL) and a negative control (DNase/RNase-free water; Zymo Research) were included in each run. No amplification was observed in the outgroup species for either the nuclear or mitochondrial targets, confirming assay specificity to Feliformia. Figure 2 demonstrates that the nuclear marker enables the quantification of nuclear DNA for the above-listed species of Feliformia without amplification of the mtDNA. Figure 3 further confirms that the assay is specific to mtDNA and nDNA target of *Lynx* spp.

Sensitivity: The analytical sensitivity of species-specific quantification was tested on serial dilutions of DNA from *F. catus*, *P. concolor*, *A. jubatus*, *C. caracal*, *P. leo*, *P. tigris*, *Lynx* sp., and *L. serval*, with DNA input ranging from 2 ng to 5 pg. Even the lowest DNA input of 5 pg provided positive results (mtDNA for *Lynx* spp., nDNA for all tested species).

Robustness: Assay robustness was assessed by varying the annealing temperature. The optimal annealing temperature was 60 °C. Deviations greater than +2 °C resulted in amplification failure, indicating the assay’s sensitivity to thermal conditions.

Repeatability: The repeatability was tested on DNA extracts from 16 different individuals (*Lynx* spp.) in triplicate. All experiments, including the negative controls, provided consistent results across replicates, demonstrating the repeatability of the assay.

Reproducibility: Two different technicians ran the assay of the same set of 10 samples independently (within-lab reproducibility). All the runs, including the negative controls, provided concordant results, confirming that the assay produces reproducible outcomes across users.

### 4.2. Llyn STRPlex

Specificity, sensitivity, robustness, repeatability, and reproducibility were assessed following the protocol for the *Llynx Qplex* assay described in the Materials and Methods Section 2.3.

Specificity: The STR-typing multiplex *Llyn STRplex* incorporates previously design STRs from *Lynx lynx, Lynx canadensis*, and *F. catus.* The *Llyn STRplex* is specific to the genus *Lynx*.

Sensitivity: The STR-typing multiplex was tested with the DNA input ranging from 1 ng to 30 pg. The resulting EPGs for *Lynx* spp. are shown in Figure 5. Even the lowest nDNA input of 30 pg provided a complete DNA profile.

Robustness: The STR-typing multiplex *Llyn STRplex* was tested under different annealing temperatures from the original 55 °C. Changes greater than +2 °C cause unbalanced peaks and/or allelic drop-outs, indicating that performance is sensitive to thermal variation.

Repeatability: The repeatability of the STR typing was tested on DNA extracts from 16 individuals (*Lynx* spp.), each tested in triplicate. All samples produced identical DNA profiles, including the positive controls (a selected individual used for all runs). The negative controls were free of detectable peaks.

Reproducibility: Two different technicians independently tested the same set of 10 samples (within-lab reproducibility). All samples provided concordant DNA profiles, including the positive controls. The negative controls were free of detectable peaks.

## 5. Discussion

Species and individual identification are crucial in forensic investigations and species protection. Individual identification based on STR multiplexes is used across many taxa in forensic applications and supports efforts to monitor populations, enforce wildlife protection laws, and manage conservation programs effectively [61,62]. A critical aspect of STR analysis is ensuring repeatability and accuracy by precisely measuring DNA concentration. Most commonly used DNA quantification methods, such as UV spectrophotometry and fluorometry, often suffer from insufficient sensitivity, can overestimate DNA concentration due to non-specific binding, and are unable to distinguish between intact and degraded DNA [53]. Consequently, insufficient DNA input can lead to allelic dropout, resulting in incomplete or incorrect genotyping [63]. Moreover, suboptimal samples often contain inhibitors like humic acid or EDTA that can interfere with PCR reactions [64]. These challenges underscore the importance of robust quantification systems and quality control measures.

In this study, we present the results of a qPCR quantitation assay tested alongside the STR-typing multiplex for *Lynx* spp. QPCR quantitation assays offer the potential of multiplexing, sufficient sensitivity for the detection of low-concentration template DNA. QPCR quantification system, *Llynx Qplex*, incorporates not only a genus-specific marker (*Cyt b*), and nuclear marker specific to Feliformia, but also an artificial internal positive control to detect inhibitors and ensure proper reaction conditions [33,35]. Inclusion of internal positive control is crucial for detection of potential inhibitors, especially for environmental and forensic samples that frequently contain PCR inhibitors that can be copurified during DNA extraction step [64]. From this perspective, qPCR provides significant advantages over conventional PCR when working with degraded or inhibitor-rich samples, as it enables the simultaneous detection of target DNA and internal controls [65,66].

**Figure 6:**
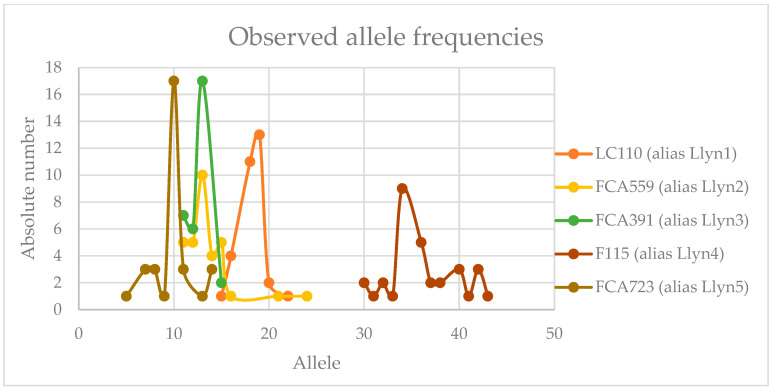
Allele frequencies (absolute number on y-axis) for particular alleles of *Llyn STRplex* loci as observed for *Lynx lynx* and *Lynx canadensis* individuals (*n* = 16).

As demonstrated by the data in Table 3 and Figure 6, the set of multiplexed STR loci (5 loci and a sex determination system) seems to be sufficient for basic individual identification. None of the *Lynx* spp. (*n* = 16) individuals tested in this study exhibited the same DNA profile (see Table 3). Although the limited sample size precluded population-level analyses or kinship calculations, the validated quantification and profiling systems offer significant potential for analyzing *Lynx* spp. samples. For instance, they allow for comparisons between reference and stain samples and enable the detection of *Lynx* spp. DNA in environmental DNA (eDNA) samples with high sensitivity. This sensitivity is sufficient even for low-yielding samples, such as feces, tanned hides, and hair.

Research targeting the identification of endangered species has a leverage effect in many areas. It not only enables law enforcement agencies to act in a timely manner but also aids in solving cold cases through database searches, including the use of rapid DNA profiling for early identification. Furthermore, progress in innovative techniques facilitates international cooperation and promotes further research in the field of wildlife forensic genetics [67,68].

## 6. Conclusions

The presented work describes the molecular tools for species-specific qPCR and STR-based individual identification of *Lynx* spp. The *Llynx Qplex* quantification system is *Lynx*-species-specific and provides information about the presence of inhibitors and reaction failure (internal positive control). The STR multiplex *Llyn STRplex* was tested on 15 unrelated individuals of *Lynx lynx* and 1 *Lynx canadensis* individual. The STR profile was not obtained for *L. rufus* due to low yields of DNA extracted (<2 pg of nDNA). The DNA-typing assay is robust and sensitive, thus suitable for forensic casework and databasing. The dataset will be expanded to develop a viable reference database for accurate individual identification and relationship testing.

## Figures and Tables

**Table 1:** Primer and probes used in *Llynx Qplex*.

Primer/Probe Name	Final Concentration (mM)	Sequence (5′-3′)	PCR Product Size (bp = Base Pairs)	Specificity	TaqMan Probe Fluorescent Label
qLynxM_F	5	GTCCCCTTCCACCCATACTAT	139 bp	*Cyt b* (mtDNA)	---
qLynxM_R	5	ACTTAGGGGGTTAGCGGGGATATAA			---
qLynxM_probe	1.7	CTCACCAGACCTGTTAGGA		probe	VIC
qLynxC_F	5	CTGCTAGGTTTAGCGCGTGAC	261 bp	IPC	---
qLynxC_R	5	GGGGACCATGCTTGCG			---
qLynx_probe	1.7	TGCACGATTCAAGCACGAT		probe	NED
qLynxN_F	3.3	AGTCCACTTCTCATTGCCCCTT	132 bp	PLP (nDNA)	---
qLynxN_R	3.3	ACCTTCCCTGAGTTCTCCATACC			---
qLynxN_probe	1.7	CTCACCAGACCTGTTAGGA		probe	6-FAM

**Table 2:** STR multiplex *Llyn STRplex*.

STR Marker	Label	Size (bp)	Repeat Motif	Primer Design	Primer Concentration (µM)
Amelogenin	FAM	193; 214		[59]	2.75
Lc110 (alias *Llyn1*)	ATTO565	91–120	(T)3(GT)14	[45]	0.75
FCA559 (alias *Llyn2*)	YAKYE	100–195	(GAAA)n	[58]	2.75
FCA391 (alias *Llyn3*)	ATTO550	129–273	(GATA)n	[58]	1
F115 (alias *Llyn4*)	ATTO565	193–250	(GAA)n	[58]	1.25
FCA723 (alias *Llyn5*)	FAM	243–317	(AAAG)n	[58]	0.75

**Table 3:** STR typing results for 16 unrelated *Lynx* spp. Individuals (15 *Lynx lynx*, 1 *Lynx canadensis*).

Species	Sample	Amelogenin	Lc110 (Alias *Llyn1*)	FCA559 (Alias *Llyn2*)	FCA391 (Alias *Llyn3*)	F115 (Alias *Llyn4*)	FCA723 (Alias *Llyn5*)
*Lynx lynx*	LL57	F	18,19	14,14	13,13	34,36	10,10
*Lynx lynx*	LL56	M	19,19	12,13	13,13	34,42	10,11
*Lynx lynx*	LL40	F	19,20	12,12	12,13	40,42	10,13
*Lynx lynx*	LL41	M	19,19	15,15	13,13	30,34	8,11
*Lynx lynx*	LL55	F	18,18	11,13	11,12	33,40	8,10
*Lynx lynx*	LL61	M	18,22	11,11	11,13	36,37	10,14
*Lynx lynx*	LL54	M	16,20	11,14	13,13	34,36	8,11
*Lynx lynx*	LL62	M	18,19	12,13	12,15	42,43	5,7
*Lynx lynx*	LL147	F	18,19	13,15	11,13	34,37	10,10
*Lynx lynx*	LL65	M	18,19	11,13	11,12	32,40	7,10
*Lynx lynx*	LL67	M	18,19	13,13	13,15	32,34	9,10
*Lynx lynx*	LL68	M	18,19	13,15	11,12	34,38	10,14
*Lynx lynx*	LL148	F	15,16	13,14	13,13	30,36	10,10
*Lynx lynx*	LL149	F	16,19	12,16	13,13	34,36	10,10
*Lynx lynx*	LL63	M	18,19	13,15	11,12	34,38	10,14
*Lynx canadensis*	LC52	M	16,18	21, 24	11,13	31,41	7,10

## Data Availability

The data presented in this study are available on request from the corresponding author due to request of the funding agency.

## References

[1] Wilson-Wilde L. (2010). Wildlife Crime: A Global Problem. Forensic Sci. Med. Pathol..

[2] Moreto W.D., Van Uhm D.P. (2021). Nested Complex Crime: Assessing the Convergence of Wildlife Trafficking, Organized Crime and Loose Criminal Networks. Br. J. Criminol..

[3] Zimmerman M.E. (2003). The Black Market for Wildlife: Combating Transnational Organized Crime in the Illegal Wildlife Trade. Vanderbilt J. Transnatl. Law.

[4] Bartlett S.E., Davidson W.S. (1991). Identification of Thunnus Tuna Species by the Polymerase Chain Reaction and Direct Sequence Analysis of Their Mitochondrial Cytochrome b Genes. Can. J. Fish. Aquat. Sci..

[5] Leeton P., Christidis L., Westerman M. (1993). Feathers from Museum Bird Skins: A Good Source of DNA for Phylogenetic Studies. Condor.

[6] Savolainen V., Cuénoud P., Spichiger R., Martinez M.D.P., Crèvecoeur M., Manen J.-F. (1995). The Use of Herbarium Specimens in DNA Phylogenetics: Evaluation and Improvement. Plant Syst. Evol..

[7] Spooner D.M., Anderson G.J., Jansen R.K. (1993). Chloroplast DNA Evidence for the Interrelationships of Tomtoes, Potatoes, and Pepinos (*Solanaceae*). Am. J. Bot..

[8] Remsen J.V. (1995). The Importance of Continued Collecting of Bird Specimens to Ornithology and Bird Conservation. Bird Conserv. Int..

[9] Ellegren H. (1992). Polymerase-Chain-Reaction (PCR) Analysis of Microsatellites: A New Approach to Studies of Genetic Relationships in Birds. Auk.

[10] Primmer C.R., Møller A.P., Ellegren H. (1995). Resolving Genetic Relationships with Microsatellite Markers: A Parentage Testing System for the Swallow *Hirundo rustica*. Mol. Ecol..

[11] Edwards S.V., Grahn M., Potts W.K. (1995). Dynamics of Mhc Evolution in Birds and Crocodilians: Amplification of Class II Genes with Degenerate Primers. Mol. Ecol..

[12] Blank R.J., Huss V.A.R. (1989). DNA Divergency and Speciation In *Symbiodinium* (*Dinophyceae*). Plant Syst. Evol..

[13] Avise J.C., Bowen B.W., Lamb T., Meylan A.B., Bermingham E. (1992). Mitochondrial DNA Evolution at a Turtle’s Pace: Evidence for Low Genetic Variability and Reduced Microevolutionary Rate in the Testudines. Mol. Biol. Evol..

[14] MacFadden B.J. (1994). Fossil Horses: Systematics, Paleobiology, and Evolution of the Family Equidae.

[15] Higuchi R.G., Wrischnik L.A., Oakes E., George M., Tong B., Wilson A.C. (1987). Mitochondrial DNA of the Extinct Quagga: Relatedness and Extent of Postmortem Change. J. Mol. Evol..

[16] Guglich E., Wilson P., White B. (1994). Forensic Application of Repetitive DNA Markers to the Species Identification of Animal Tissues. J. Forensic Sci..

[17] Cronin M.A., Palmisciano D.A., Vyse E.R., Cameron D.G. (1991). Mitochondrial DNA in Wildlife Forensic Science: Species Identification of Tissues. Wildl. Soc. Bull..

[18] Alford R.L., Caskey C.T. (1994). DNA Analysis in Forensics, Disease and Animal/Plant Identification. Curr. Opin. Biotechnol..

[19] Hermans I.F., Atkinson J., Hamilton J.F., Chambers G.K. (1991). Three Cases of Disputed Paternity in Dogs Resolved by the Use of DNA Fingerprinting. N. Z. Vet. J..

[20] Yoon C.K. (1993). Botanical Witness for the Prosecution. Science.

[21] Marklund S., Sandberg K., Andersson L. (1996). Forensic Tracing of Horse Identities Using Urine Samples and DNA Markers. Anim. Biotechnol..

[22] Dayton M., Koskinen M.T., Tom B.K., Mattila A.-M., Johnston E., Halverson J., Fantin D., DeNise S., Budowle B., Smith D.G. (2009). Developmental Validation of Short Tandem Repeat Reagent Kit for Forensic DNA Profiling of Canine Biological Materials. Croat. Med. J..

[23] Butler J.M., David V.A., O’Brien S.J., Menotti-Raymond M. (2002). The MeowPlex: A New DNA Test Using Tetranucleotide STR Markers for the Domestic Cat. Profiles DNA.

[24] Imaizumi K., Akutsu T., Miyasaka S., Yoshino M. (2007). Development of Species Identification Tests Targeting the 16S Ribosomal RNA Coding Region in Mitochondrial DNA. Int. J. Leg. Med..

[25] Budowle B., Garofano P., Hellman A., Ketchum M., Kanthaswamy S., Parson W., van Haeringen W., Fain S., Broad T. (2005). Recommendations for Animal DNA Forensic and Identity Testing. Int. J. Leg. Med..

[26] Linacre A., Gusmão L., Hecht W., Hellmann A.P., Mayr W.R., Parson W., Prinz M., Schneider P.M., Morling N. (2011). ISFG: Recommendations Regarding the Use of Non-Human (Animal) DNA in Forensic Genetic Investigations. Forensic Sci. Int. Genet..

[27] Harper C.K. (2021). RhODIS^®^ (The Rhinoceros DNA Index System): The Application of Simple Forensic and Genetic Tools Help Conserve African Rhinoceros. Wildlife Biodiversity Conservation.

[28] Harper C.K., Vermeulen G.J., Clarke A.B., de Wet J.I., Guthrie A.J. (2013). Extraction of Nuclear DNA from Rhinoceros Horn and Characterization of DNA Profiling Systems for White (*Ceratotherium simum*) and Black (*Diceros bicornis*) Rhinoceros. Forensic Sci. Int. Genet..

[29] Wasser S.K., Mailand C., Booth R., Mutayoba B., Kisamo E., Clark B., Stephens M. (2007). Using DNA to Track the Origin of the Largest Ivory Seizure since the 1989 Trade Ban. Proc. Natl. Acad. Sci. USA.

[30] Wasser S.K., Brown L., Mailand C., Mondol S., Clark W., Laurie C., Weir B.S. (2015). Genetic Assignment of Large Seizures of Elephant Ivory Reveals Africa’s Major Poaching Hotspots. Science.

[31] Wasser S.K., Clark W.J., Drori O., Kisamo S.E., Mailand C., Mutayoba B., Stephens M. (2008). Combating the Illegal Trade in African Elephant Ivory with DNA Forensics. Conserv. Biol..

[32] Wasser S.K., Wolock C.J., Kuhner M.K., Brown J.E., Morris C., Horwitz R.J., Wong A., Fernandez C.J., Otiende M.Y., Hoareau Y. (2022). Elephant Genotypes Reveal the Size and Connectivity of Transnational Ivory Traffickers. Nat. Hum. Behav..

[33] Vankova L., Vanek D. (2022). DNA-Based Identification of Big Cats and Traditional Chinese Medicine Artifacts in the Czech Republic. Forensic Sci. Int. Genet. Suppl. Ser..

[34] Vankova L., Vanek D. (2024). Capillary-Electrophoresis-Based Species Barcoding of Big Cats: CR-MtDNA-Length Polymorphism. Life.

[35] Vaněk D., Ehler E., Vaňková L. (2021). Technical Note: Development of DNA Quantitation and STR Typing Systems for *Panthera Tigris* Species Determination and Individual Identification in Forensic Casework. Eur. J. Environ. Sci..

[36] Hebenstreitova K., Salaba O., Trubac J., Kufnerova J., Vanek D. (2024). The Influence of Tanning Chemical Agents on DNA Degradation: A Robust Procedure for the Analysis of Tanned Animal Hide—A Pilot Study. Life.

[37] Morf N.V., Kopps A.M., Nater A., Lendvay B., Vasiljevic N., Webster L.M.I., Fautley R.G., Ogden R., Kratzer A. (2021). STRoe Deer: A Validated Forensic STR Profiling System for the European Roe Deer (*Capreolus capreolus*). Forensic Sci. Int. Anim. Environ..

[38] Meredith E.P., Adkins J.K., Rodzen J.A. (2020). UrsaPlex: An STR Multiplex for Forensic Identification of North American Black Bear (*Ursus americanus*). Forensic Sci. Int. Genet..

[39] Friedenberger A., Doyle C., Couillard L., Kyle C.J. (2023). The Bear Necessities: A Sensitive QPCR Assay for Bear DNA Detection from Bile and Derived Products to Complement Wildlife Forensic Enforcement. Forensic Sci. Int. Genet..

[40] Hrebianchuk A.E., Parfionava N.S., Zabauskaya T.V., Tsybovsky I.S. (2024). A Panel of Tetranucleotide STR Markers as an Alternative Approach to Forensic DNA Identification of Wolf and Dog. Anim. Genet..

[41] Berger B., Berger C., Hecht W., Hellmann A., Rohleder U., Schleenbecker U., Parson W. (2014). Validation of Two Canine STR Multiplex-Assays Following the ISFG Recommendations for Non-Human DNA Analysis. Forensic Sci. Int. Genet..

[42] Selkoe K.A., Toonen R.J. (2006). Microsatellites for Ecologists: A Practical Guide to Using and Evaluating Microsatellite Markers. Ecol. Lett..

[43] Rueness E.K., Jorde P.E., Hellborg L., Stenseth N.C., Ellegren H., Jakobsen K.S. (2003). Cryptic Population Structure in a Large, Mobile Mammalian Predator: The Scandinavian Lynx. Mol. Ecol..

[44] Herrero A., Klütsch C.F.C., Holmala K., Maduna S.N., Kopatz A., Eiken H.G., Hagen S.B. (2021). Genetic Analysis Indicates Spatial-Dependent Patterns of Sex-Biased Dispersal in Eurasian Lynx in Finland. PLoS ONE.

[45] Carmichael L.E., Clark W., Strobeck C. (2000). Development and Characterization of Microsatellite Loci from Lynx (*Lynx canadensis*), and Their Use in Other Felids. Mol. Ecol..

[46] Krojerová-Prokešová J., Turbaková B., Jelenčič M., Bojda M., Kutal M., Skrbinšek T., Koubek P., Bryja J. (2019). Genetic Constraints of Population Expansion of the Carpathian Lynx at the Western Edge of Its Native Distribution Range in Central Europe. Heredity.

[47] Gajdárová B., Belotti E., Bufka L., Volfová J., Wölfl S., Mináriková T., Hollerbach L., Duľa M., Kleven O., Kutal M. (2023). Long-Term Genetic Monitoring of a Reintroduced Eurasian Lynx Population Does Not Indicate an Ongoing Loss of Genetic Diversity. Glob. Ecol. Conserv..

[48] Janečka J.E., Blankenship T.L., Hirth D.H., Tewes M.E., Kilpatrick C.W., Grassman L.I. (2006). Kinship and Social Structure of Bobcats (*Lynx rufus*) Inferred from Microsatellite and Radio-telemetry Data. J. Zool..

[49] Palormes F., Godoy J.A., López-Bao J.V., Rodríguez A., Roques S., Casas-Marce M., Revilla E., Delibes M. (2012). Possible Extinction Vortex for a Population of Iberian Lynx on the Verge of Extirpation. Conserv. Biol..

[50] Jun J., Han S.H., Jeong T.-J., Park H.C., Lee B., Kwak M. (2011). Wildlife Forensics Using Mitochondrial DNA Sequences: Species Identification Based on Hairs Collected in the Field and Confiscated Tanned Felidae Leathers. Genes Genom..

[51] Waits L.P., Paetkau D. (2005). Non-Invasive Genetic Sampling Tools for Wildlife Biologists: A Review of Applications and Recommendations for Accurate Data Collection. J. Wildl. Manag..

[52] Simbolo M., Gottardi M., Corbo V., Fassan M., Mafficini A., Malpeli G., Lawlor R.T., Scarpa A. (2013). DNA Qualification Workflow for Next Generation Sequencing of Histopathological Samples. PLoS ONE.

[53] Lee S.B., McCord B., Buel E. (2014). Advances in Forensic DNA Quantification: A Review. Electrophoresis.

[54] Červený J., Koubek P., Bufka L. (2002). Eurasian Lynx (*Lynx lynx*) and Its Chance for Survival in Central Europe: The Case of the Czech Republic. Acta Zool. Litu..

[55] Schmidt K., Ratkiewicz M., Konopinski M.K. (2011). The Importance of Genetic Variability and Population Differentiation in the Eurasian Lynx *Lynx lynx* for Conservation, in the Context of Habitat and Climate Change. Mammal Rev..

[56] Sommer R.S., Benecke N. (2006). Late Pleistocene and Holocene Development of the Felid Fauna (*Felidae*) of Europe: A Review. J. Zool..

[57] Arlettaz R., Chapron G., Kéry M., Klaus E., Mettaz S., Roder S., Vignali S., Zimmermann F., Braunisch V. (2021). Poaching Threatens the Establishment of a Lynx Population, Highlighting the Need for a Centralized Judiciary Approach. Front. Conserv. Sci..

[58] Menotti-Raymond M., David V.A., Lyons L.A., Schäffer A.A., Tomlin J.F., Hutton M.K., O’Brien S.J. (1999). A Genetic Linkage Map of Microsatellites in the Domestic Cat (*Felis catus*). Genomics.

[59] Pilgrim K.L., McKeley K.S., Riddle A.E., Schwartz M.K. (2005). Felid Sex Identification Based on Noninvasive Genetic Samples. Mol. Ecol. Notes.

[60] Ye J., Coulouris G., Zaretskaya I., Cutcutache I., Rozen S., Madden T.L. (2012). Primer-BLAST: A Tool to Design Target-Specific Primers for Polymerase Chain Reaction. BMC Bioinform..

[61] Ng K.K.S., Lee S.L., Tnah L.H., Nurul-Farhanah Z., Ng C.H., Lee C.T., Tani N., Diway B., Lai P.S., Khoo E. (2016). Forensic Timber Identification: A Case Study of a CITES Listed Species, *Gonystylus bancanus* (Thymelaeaceae). Forensic Sci. Int. Genet..

[62] Potoczniak M.J., Chermak M., Quarino L., Tobe S.S., Conte J. (2020). Development of a Multiplex, PCR-Based Genotyping Assay for African and Asian Elephants for Forensic Purposes. Int. J. Leg. Med..

[63] Gill P., Whitaker J., Flaxman C., Brown N., Buckleton J. (2000). An Investigation of the Rigor of Interpretation Rules for STRs Derived from Less than 100 Pg of DNA. Forensic Sci. Int..

[64] Schrader C., Schielke A., Ellerbroek L., Johne R. (2012). PCR Inhibitors—Occurrence, Properties and Removal. J. Appl. Microbiol..

[65] Sidstedt M., Jansson L., Nilsson E., Noppa L., Forsman M., Rådström P., Hedman J. (2015). Humic Substances Cause Fluorescence Inhibition in Real-Time Polymerase Chain Reaction. Anal. Biochem..

[66] Sidstedt M., Rådström P., Hedman J. (2020). PCR Inhibition in QPCR, DPCR and MPS—Mechanisms and Solutions. Anal. Bioanal. Chem..

[67] Polner M., Moell D. (2016). Interagency Collaboration and Combating Wildlife Crime. Environmental Crime and Collaborative State Intervention.

[68] Van Asch E. (2017). Exploring the Effectiveness of International Cooperation to Combat Transnational Organized Wildlife Crime: Lessons Learned from Initiatives in Asia. Ph.D. Thesis.

